# Sustained Release of Prostaglandin E_**2**_ in Fibroblasts Expressing Ectopically Cyclooxygenase 2 Impairs P2Y-Dependent Ca^**2+**^-Mobilization

**DOI:** 10.1155/2014/832103

**Published:** 2014-08-18

**Authors:** María Pimentel-Santillana, Paqui G. Través, Raquel Pérez-Sen, Esmerilda G. Delicado, Paloma Martín-Sanz, María Teresa Miras-Portugal, Lisardo Boscá

**Affiliations:** ^1^Instituto de Investigaciones Biomédicas Alberto Sols, Centro Mixto CSIC-UAM, Arturo Duperier 4, 28029 Madrid, Spain; ^2^The Salk Institute, 10010 N Torrey Pines Road, La Jolla, CA 92037, USA; ^3^Departamento de Bioquímica y Biología Molecular IV, Facultad de Veterinaria e Instituto Universitario de Investigación en Neuroquímica, Instituto de Investigación Sanitaria del Hospital Clínico San Carlos (IdISSC), Universidad Complutense, Madrid, Spain; ^4^Centro de Investigación Biomédica en Red de Enfermedades Hepáticas y Digestivas (Ciberehd), Spain

## Abstract

The nucleotide uridine trisphosphate (UTP) released to the extracellular milieu acts as a signaling molecule *via* activation of specific pyrimidine receptors (P2Y). P2Y receptors are G protein-coupled receptors expressed in many cell types. These receptors mediate several cell responses and they are involved in intracellular calcium mobilization. We investigated the role of the prostanoid PGE_2_ in P2Y signaling in mouse embryonic fibroblasts (MEFs), since these cells are involved in different ontogenic and physiopathological processes, among them is tissue repair following proinflammatory activation. Interestingly, Ca^2+^-mobilization induced by UTP-dependent P2Y activation was reduced by PGE_2_ when this prostanoid was produced by MEFs transfected with COX-2 or when PGE_2_ was added exogenously to the culture medium. This Ca^2+^-mobilization was important for the activation of different metabolic pathways in fibroblasts. Moreover, inhibition of COX-2 with selective coxibs prevented UTP-dependent P2Y activation in these cells. The inhibition of P2Y responses by PGE_2_ involves the activation of PKCs and PKD, a response that can be suppressed after pharmacological inhibition of these protein kinases. In addition to this, PGE_2_ reduces the fibroblast migration induced by P2Y-agonists such as UTP. Taken together, these data demonstrate that PGE_2_ is involved in the regulation of P2Y signaling in these cells.

## 1. Introduction

P2 receptors are purinergic receptors selective for adenosine 5′-trisphosphate (ATP), adenosine 5′-diphosphate (ADP), uridine 5′-trisphosphate (UTP), and uridine 5′-diphosphate (UDP). These nucleotides act as extracellular signaling molecules and exert their activity by binding to and activating specific membrane receptors, designed P2 receptors [1, 2]. There are two families of P2 receptors structurally distinct: P2X ionotropic ion channel receptors and P2Y metabotropic G protein-coupled receptors [3–5]. Currently, seven P2X subtypes and eight P2Y receptor subtypes are recognized, including receptors that are sensitive to pyrimidines as well as to purines [6]. Receptors for purine and pyrimidine nucleotides are involved in many neuronal as well as nonneuronal mechanisms, including short-term purinergic signaling such as neurotransmission, neuromodulation, neurosecretion, immune responses, inflammation, platelet aggregation, and vasodilatation, and long-term purinergic signaling of cell proliferation, differentiation, motility, and death in development and regeneration [7].

At present, there are eight accepted P2Y receptors: P2Y1, P2Y2, P2Y4, P2Y6, P2Y11, P2Y12, P2Y13, and P2Y14 [8, 9]. The metabotropic receptors, coupled to phospholipase C (PLC), can be activated by different nucleotides, depending on the P2Y receptors and the species studied [10]. In the aftermath of nucleotide release to the extracellular medium these receptors are stimulated leading to an intracellular increase in diacylglycerol (DAG) and inositol trisphosphate (IP3) followed by a release of calcium from intracellular stores [11, 12]. These receptors are widely distributed in several cell types; their existence in fibroblasts was first reported by Okada et al. [13]. Fibroblasts respond to inflammation and damage, being involved in the repair phase following tissue damage, or in other pathological circumstances, such as atherogenesis [14].

Prostaglandin E_2_ (PGE_2_) is an important chemical mediator generated from arachidonic acid* via* the cyclooxygenase pathway. The various biological effects of PGE_2_ are mediated by four receptors called E-type prostanoid receptors (EP1 to EP4), which are G protein-coupled membrane receptors [15]. EP1 leads to mobilization of intracellular calcium. This transient change in intracellular Ca^2+^ alters the activity of many proteins, including several isoforms of PKC. Therefore, PGE_2_ evokes Ca^2+^- and PKC-mediated effects in cells expressing EP1 [16]. EP2 and EP4 signaling generates increased intracellular cyclic AMP (cAMP) levels, whereas EP3 leads to a reduction in intracellular cAMP levels [17, 18]. However, in addition to EP-mediated effects, PGs may exert other EP-independent actions, for example, through the purinergic signaling [19, 20].

Taken together, both signaling pathways generate DAG and IP3, promoting Ca^2+^ mobilization. This alteration may affect the activity of several proteins, such as PKC and, indeed, previous work have described that the signaling of G protein-coupled receptors is regulated by mechanisms involving protein kinases such as PKC [21]. Although it has been shown that PGE_2_ is a potent inhibitor of the purinergic signaling mediated by some purinergic receptors [19, 20], less is known about the underlying cross-talk between PGs and P2 signaling as a mechanism integrating inflammation and the presence of extracellular nucleotides. In the present work we have investigated this interplay between PGs and P2 receptors in mouse embryonic fibroblasts (MEFs). Our data extend previous work in macrophages and suggest that this communication between the two pathways is functional in MEFs adding a new piece of knowledge to understand how fibroblast activity may be regulated by these dual signaling pathways.

## 2. Materials and Methods

### 2.1. Reagents

UTP, ionophores, and standard reagents were from Sigma-Aldrich (St Louis, MO, USA). DFU was from Merck (Rahway, NJ, USA). Prostaglandin E_2_ was from Cayman Chemical (Ann Arbor, MI, USA). Gö6976, Gö6983, Gö6850, and inhibitors of standard signaling pathways were from Calbiochem (San Diego, CA, USA). Fura-2/AM was from Invitrogen (Carlsbad, CA, USA). Cytokines were from PeproTech (London, UK). Antibodies against P2Y2, P2Y4, and P2Y6 receptors were from Alomone Labs (Jerusalem, Israel) and other antibodies were from Santa Cruz Biotech (Santa Cruz, CA, USA), from Cell Signaling (Danvers, MA, USA), or from the sources previously described [22]. Reagents for electrophoresis were from Bio-Rad (Hercules, CA, USA) and Sigma-Aldrich. Tissue culture dishes were from Falcon (Lincoln Park, NJ, USA) and culture media were from Invitrogen.

### 2.2. Animals

COX-2 wild type (WT) and COX-2-deficient mice, with a mixed background 129SV and C57BL/6, were obtained from the Jackson Laboratory. Mice were housed under 12 h light/dark cycle and food and water were provided* ad libitum*. Animals were cared for according to a protocol approved by the Ethical Committee of our institution (following directive 2010/63/EU of the European Parliament).

### 2.3. Preparation of Mouse Embryonic Fibroblasts (MEFs), Cell Culture, and MEFs Immortalization

MEFs were prepared from E14.5 embryos from WT and COX-2-deficient mice. Briefly, female mice were euthanized by CO_2_ at 14.5 after conception. Using scissors, the abdomen was opened and the embryos were isolated with their yolk sacs intact. The yolk sac was removed and retained for genotyping. The head and internal organs of each embryo were discarded. The dissected embryo was passed through an 18G needle to disperse the cells [23]. MEFs were cultured (2 × 10^6^ cells per 60 mm dish or in 12-multiwell plates at a density of 2 × 10^5^ cells/well) in Dulbecco's Modified Eagle's Medium (DMEM) supplemented with 10% fetal bovine serum (FBS), 100 U/mL penicillin, 100 *μ*g/mL streptomycin at 37°C, and 5% CO_2_ [24, 25]. COX-2^+/+^ and COX-2^−/−^ primary MEFs were transfected with a SV40 large T-antigen expression vector using Lipofectamine 2000 (Invitrogen), according to manufacturer's instructions, to obtain immortalized MEF cell line (referred to as MEFs WT, KO, or KI-carrying the COX-2 transgene).

### 2.4. Transfection

To ectopically express COX-2, COX-2-deficient MEFs were transiently transfected with 4 *μ*g of plasmid DNA (per well in a 6-well plate) using Lipofectamine 2000 reagent following the instructions of the supplier. Briefly, MEF cells at 70% confluence were exposed for 6–16 h to Lipofectamine reagent containing pPyCAGIP-COX-2 or control vector pPyCAGIP. At the end of this period, the transfection media were replaced with fresh medium containing 10% FBS. COX-2 expression was determined by Western blot.

### 2.5. Determination of PGE_2_ by Enzyme Immunoassay

PGE_2_ accumulation was measured in the culture medium. For the assay, WT and KI (COX-2-deficient MEFs overexpressing COX-2) MEFs were plated in 6-well plates at a density of 1.5 × 10^6^ cells/well in 2 mL DMEM and treated in the absence or presence of LPS (200 ng/mL) plus cytokines (IFN-*γ*, TNF-*α*, and IL-1*β*, 20 ng/mL) for 18 h at 37°C. The culture supernatants were centrifuged at 12,000 ×g for 5 min and PGE_2_ levels were determined by specific immunoassay (DetectX Prostaglandin E_2_, Arbor Assays, Ann Arbor MI, USA), according to the manufacturer's instructions.

### 2.6. Calcium Dynamic Analysis

MEFs attached to coverslips were incubated in Locke's solution as previously described [20]. The effect of purinergic receptor agonists was assayed at near-maximal effective concentrations (100 *μ*M UTP) [26]. In other studies, 5 *μ*M PGE_2_ was applied for 5 min before nucleotides perfusion in the presence of prostanoids. When pharmacological inhibitors were used, they were preincubated at the indicated concentrations and for the required times as specified in the text and figure legends and kept during prostanoid incubation and/or purinergic agonist stimulation. After dual excitation at 340 and 380 nm the fluorescence was recorded and analyzed. Background signals were subtracted from each wavelength and the F_340_/F_380_ ratio was calculated [27]. Alternatively, in some cases (indicated in the corresponding figure legends), calcium mobilization was measured using the nonratiometric Fluo-4 direct probe (Invitrogen), following the instructions of the supplier. In this case, the changes in fluorescence were measured in a fluorescence microscope (Observer Z1, Plan Apochromat objective, Zeiss) equipped with a Cascade1K camera, analyzed using the Axiovision 4.8 imaging program and expressed as the percentage of responding cells. Video imaging of the calcium-dependent fluorescence fluxes was also acquired.

### 2.7. RNA Isolation and Quantitative PCR (qPCR) Analysis

1 *μ*g of total RNA, extracted with TRI Reagent (Ambion, Life Technologies), was reverse transcribed using Transcriptor First Strand cDNA Synthesis Kit for RT-PCR following the indications of the manufacturer (Roche). Real-time PCR was conducted with SYBR Green (Roche) on a MyiQ Real-Time PCR System (Bio-Rad). The TaqMan probes for mouse EP1, EP2, EP3, EP4, P2Y2, and P2Y4 used in this study were purchased from Applied Biosystems and experiments for validation of amplification efficiency were performed for each TaqMan probe set [28, 29]. PCR thermocycling parameters were 95°C for 10 min, 40 cycles of 95°C for 15 s, and 60°C for 1 min. Each sample was run in duplicate and was normalized with the expression of 36B4. The fold induction (FI) was determined in a ΔΔCt based fold-change calculations (relative quantity, RQ, is 2^−ΔΔCt^).

### 2.8. Preparation of Total Protein Cell Extracts and Western Blot Analysis

Cells were homogenized in a buffer containing 10 mM Tris-HCl, pH 7.5; 1 mM MgCl_2_, 1 mM EGTA, 10% glycerol, 0.5% CHAPS, 1 mM *β*-mercaptoethanol, and a protease and phosphatase inhibitor cocktail (Sigma). The extracts were vortexed for 30 min at 4°C and after centrifuging for 15 min at 13,000 ×g, the supernatants were stored at −20°C. Protein levels were determined with Bradford reagent (Bio-Rad). For immunoblot analysis the protein extracts were analyzed as described using a Charged Coupling Device camera in a luminescent image analyzer (Molecular Imager, BioRad) to ensure the linearity of the band intensities. Values of densitometry were determined using Quantity One software (Bio-Rad).

### 2.9. MEFs Migration in Transwells

Migration assays were performed in 24 transwells (uncoated 8 *μ*m porous transwells) according to the manufacturer's instructions (Corning Incorporated, NY). 5 × 10^4^ MEFs were seeded in the upper chambers and cells were allowed to attach for 2 h. After thorough washing with PBS to remove nonadherent cells, MEFs were starved overnight. Cells were stimulated with combinations of the indicated stimuli (PGE_2_ and UTP in the upper chamber and 10% FBS, UTP, or PGE_2_ into 500 *μ*L in the lower chamber, used as chemoattractants). The plates were incubated at 37°C overnight in the presence of 20 *μ*g/mL of mitomycin C (Sigma-Aldrich) to inhibit cell proliferation. The membrane was fixed with paraformaldehyde (4%; pH 7.2) and stained with crystal violet solution (Sigma-Aldrich). The number of cells that migrated completely through the 8 *μ*m pores was determined.

### 2.10. Statistical Analysis

The values in graphs correspond to the mean ± SD. The statistical significance was estimated with a Student's *t*-test for unpaired observation. Data were analyzed by the SPSS for Windows statistical package, version 21.

## 3. Results

### 3.1. Transgenic Expression of COX-2 Impairs P2Y-Dependent Ca^2+^-Mobilization

**Figure 1:**
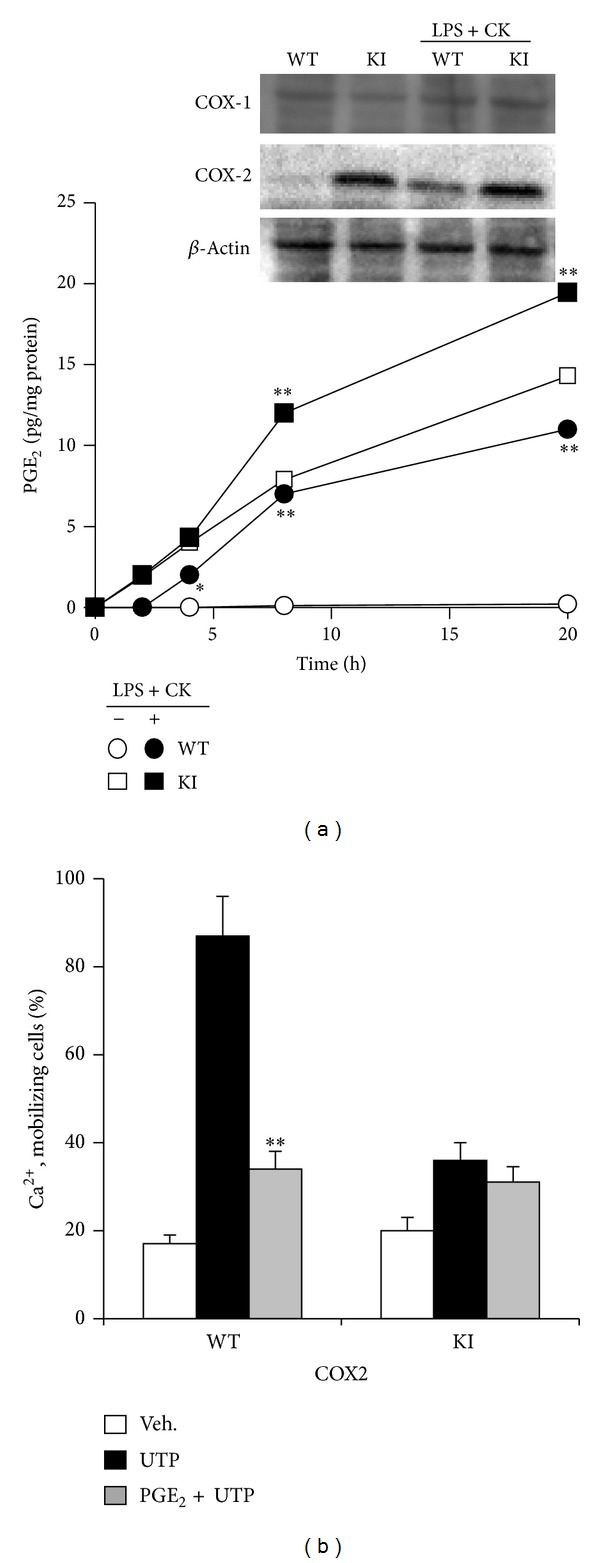
PGE_2_ released in MEFs overexpressing COX-2 and effect on P2Y-dependent Ca^2+^-mobilization. WT and KI (COX-2-deficient MEFs overexpressing COX-2) MEFs, treated in the absence or presence of LPS (200 ng/mL) plus cytokines (IFN-*γ*, TNF-*α*, and IL-1*β*, 20 ng/mL), were used. The protein levels of COX-1 and COX-2 and the PGE_2_ released into the culture medium were determined by immunoblot and ELISA, respectively (a). The percentage of cells showing Ca^2+^-mobilization in response to the P2Y agonist UTP (100 *μ*M) was determined using the nonratiometric Fluo-4 assay (b). Results show a representative blot (a) and the mean + SD of three experiments for release of PGE_2_ to the culture medium and Ca^2+^-mobilization. *P < 0.05, **P < 0.001 versus the corresponding control.

**Figure 2:**
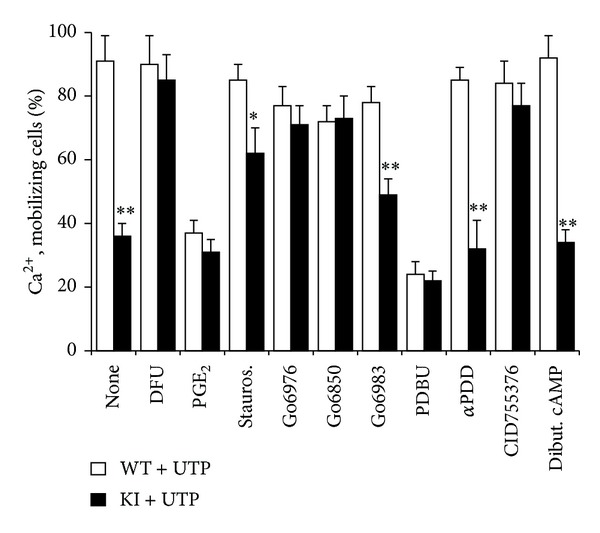
Characterization of targeting of PKC, PKD, and PKA on the effect of PGE_2_ on the UTP-dependent Ca^2+^-mobilization. WT or KI MEFs were washed with fresh medium and maintained in culture for 1 h to remove PGE_2_ accumulated and then treated for 10 min with the indicated effectors, except for DFU that was added immediately after washing (1 *μ*M DFU, an inhibitor of COX-2; 5 *μ*M PGE_2_; 100 nM staurosporine; 100 nM Gö6976; 5 *μ*M Gö6850; 10 nM Gö6983, a selective inhibitor of classic PKCs; 200 nM PDBU; 200 nM *α*PDD; 200 nM CID755376, a selective inhibitor of PKD; 5 *μ*M dibutyryl cAMP) and the percentage of cells showing Ca^2+^-mobilization in response to UTP (100 *μ*M) was determined using the nonratiometric Fluo-4 assay. Results show the mean + SD of three experiments for Ca^2+^-mobilization. *P < 0.05, **P < 0.001 versus the same condition in the WT cells.

MEFs expressing COX-2 release PGE_2_ in the absence of additional stimuli. This accumulation was enhanced after proinflammatory stimulation with a combination of LPS, TNF-*α*, IL-1*β*, and IFN-*γ* (Figure 1(a)). In addition to this, the presence of PGE_2_ inhibited UTP-dependent Ca^2+^-mobilization in MEF cells, either when this PG is exogenously added or when produced by the COX-2 transgene (Figure 1(b)). A video imaging of the Ca^2+^-transients in COX-2 WT and KI (expressing the COX-2 transgene) MEFs treated with different stimuli (PGs and UTP) is shown in Supplementary Figure S1 in Supplementary Material available online at http://dx.doi.org/10.1155/2014/832103. Interestingly, when the medium of COX-2 KI MEFs is replaced by fresh medium containing the selective COX-2 inhibitor DFU (a coxib), the impaired P2Y signaling in response to UTP observed in the same cells without medium change was abolished (Figure S1). Analysis of the pathways involved in the impairment of P2Y-dependent Ca^2+^-mobilization in MEFs from WT and COX-2 transfected cells (KI) showed a similar pattern of responses between both conditions for exogenous PGE_2_ or when COX-2 was inhibited with DFU (Figure 2). The UTP mobilization of Ca^2+^ was similar between MEFs from WT or COX-2-deficient mice (data not shown), regardless of the treatment with DFU. In addition to this, a broad inhibitor of PKCs and some tyrosine kinases (staurosporine) partially rescued the response to UTP in KI cells. Interestingly, inhibition of novel PKCs (*δ*, *ε*, *η*, and *θ*) [30] and PKD (Gö6976 and CID755376, resp.), but not of the classic isoforms of PKC (*α*, *β*, and *γ*; inhibited with Gö6983), restored the response to UTP in the presence of PGE_2_ due to the activity of COX-2. Opposite to this, activation of PKCs/PKD with the diacylglycerol analogue PDBu abolished the UTP-dependent Ca^2+^-mobilization, whereas the inactive phorbol *α*-PDD was unable to influence the responses of both types of cells. PKA activation after treatment with a permeant cAMP analogue (dibutyril-cAMP) was also unable to affect UTP signaling (Figure 2). Together, these results suggest that novel PKCs and PKD are involved in the suppressive effect of PGE_2_ on UTP-dependent Ca^2+^-mobilization.

### 3.2. Transgenic Expression of COX-2 Accumulates P2Y4 Receptors in the Nucleus

**Figure 3:**
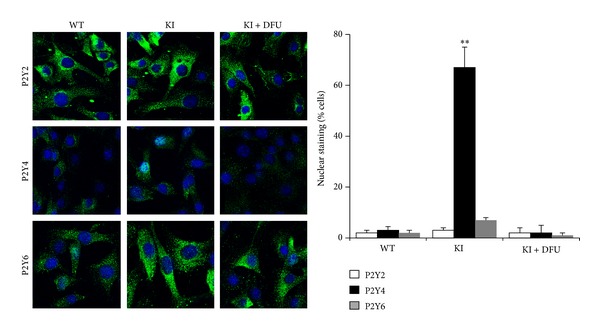
Subcellular distribution of P2Y2, P2Y4, and P2Y6 receptors in MEFs. WT or KI MEFs were cultured and, after changing the medium, were maintained in the absence or presence of 1 *μ*M DFU for 2 h. Cells were fixed with paraformaldehyde (4%; pH 7.2) and permeabilized with cold methanol at RT. After incubation with anti-P2Y2, anti-P2Y4 and anti-P2Y6 antibodies (1 : 500) overnight at 4°C, cells were visualized by confocal microscopy using a FITC-conjugated secondary Ab (Alexa-Fluor 488, 1 : 1000). Nuclei were stained with Hoechst 33258. Coverslips were mounted in Prolong Gold antifade reagent (Molecular Probes) and the intensity of the fluorescence was measured using Image J software (NIH, Bethesda, MD, USA). Results show the mean + SD of three experiments. **P < 0.001 versus the same condition in the WT cells.

**Figure 4:**
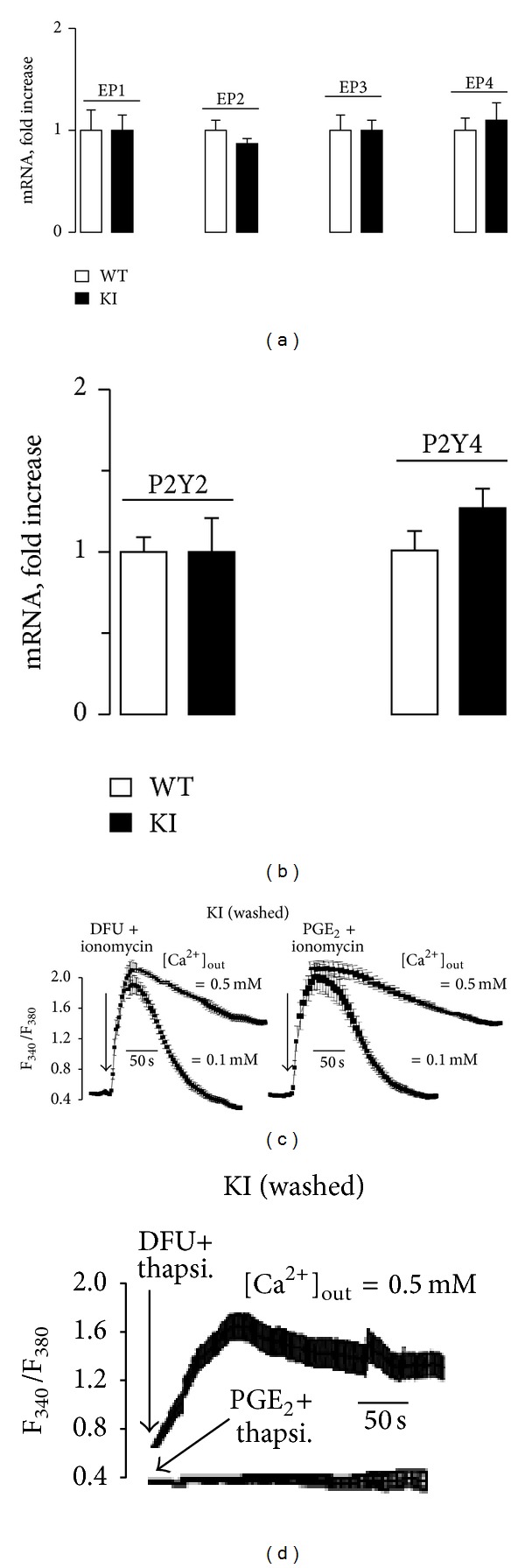
Characterization of EP1–4 and P2Y2–P2Y4 expression and effect of ionophores on Ca^2+^-mobilization in MEF cells. The expression levels of the prostaglandin receptors EP1–4 and the levels of P2Y2 and P2Y4 were determined by qPCR (a-b). The response to 1 *μ*M ionomycin (c) and 500 nM thapsigargin (d) on Ca^2+^-mobilization was determined in MEFs overexpressing COX-2, using the dual excitation 340/380 nm protocol as described in Section 2. MEFs KI were washed with fresh medium to remove PGE_2_ accumulated and maintained in the absence or presence of 1 *μ*M DFU and 5 *μ*M PGE_2_. Different extracellular concentrations of calcium were used. Results show the mean + SD of three experiments (a-b) or a representative trace (c-d). *P < 0.05 versus the same condition in WT cells.

To identify mechanisms involved in the impairment of P2Y signaling the distribution of these receptors in MEF cells constitutively synthesizing PGE_2_ was analyzed. As Figure 3 shows, P2Y2, P2Y4, and P2Y6 were present in these cells; however, a significant proportion of P2Y4 receptors localized in the nucleus, a situation that was suppressed after inhibition of COX-2 with DFU. This was also observed in WT MEFs treated with PGE_2_ (Supplementary Figure S2). Interestingly, the expression of EP1–4 PGE_2_ receptors and P2Y2 and P2Y4 receptors was not influenced by the ectopic expression of COX-2 (Figures 4(a) and 4(b)). To further investigate the effect of PGE_2_ on Ca^2+^-mobilization, treatment of KI MEFs with the Ca^2+^ ionophore ionomycin resulted in identical calcium fluxes regardless of the incubation with DFU or PGE_2_ (Figure 4(c)). Indeed, the shape of the Ca^2+^ fluxes exhibited similar profiles when the extracellular Ca^2+^ concentration was maintained high (0.5 mM) or low (0.1 mM). However, Ca^2+^-mobilization in response to thapsigargin (i.e., after inhibition of the replenishment of the ER stores) was significantly inhibited in the presence of PGE_2_ (Figure 4(d)). This latter condition was similar to the mobilization induced by thapsigargin in KI cells in the absence of medium replacement (i.e., with accumulation of PGE_2_ in the culture medium, not shown).

### 3.3. Thapsigargin-Dependent Phosphorylation in MEFs

**Figure 5:**
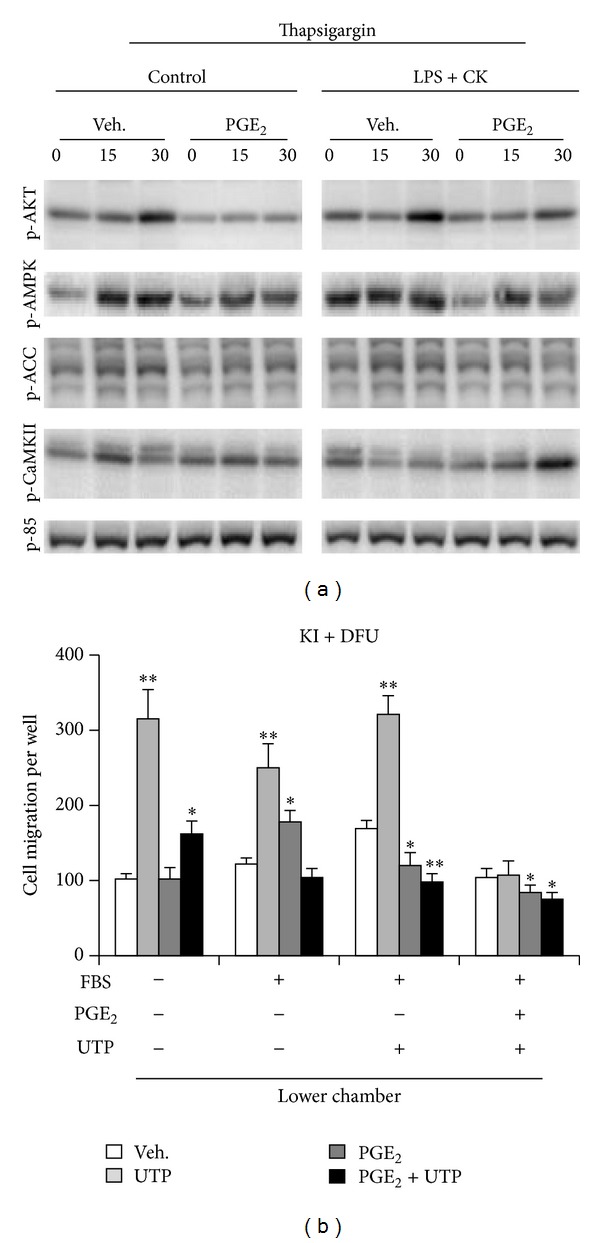
Effect of thapsigargin on Ca^2+^-mobilization and migration of MEFs in response to UTP and chemotactic stimuli. WT or COX-2 KI cells were activated, or not, for 24 h with LPS (200 ng/mL) plus cytokines (IFN-*γ*, TNF-*α*, and IL-1*β*, 20 ng/mL) and then treated for 5 min with 500 nM thapsigargin and in the absence or presence of 5 *μ*M PGE_2_. The levels of the indicated phosphoproteins were determined by Western blot (a). The capacity of these cells to migrate in transwell was determined after incubation with 5 *μ*M PGE_2_ and/or 100 *μ*M UTP. The migration was measured after 24 h of incubation in the absence or presence of different combinations of 10% FBS, PGE_2_ (5 *μ*M), or UTP (100 nM) in the lower wells (b). Results show a representative blot (a) out of three or the mean + SD of four experiments (b). *P < 0.05; **P < 0.001 versus the same condition in the absence of treatment in the upper chamber.

To evaluate the effect of PGE_2_ on Ca^2+^-mobilization, cells were treated with prostaglandin and immediately with thapsigargin. As Figure 5(a) shows, the phosphorylation of AKT was inhibited by PGE_2_ and to lesser extents in a proinflammatory situation. Similar results were obtained for the phosphorylation of CaMKII and ACC, whereas AMPK phosphorylation was minimally affected by PGE_2_. These data suggest a complex pattern of phosphorylation of these enzymes beyond P2Y activation, as previously described in macrophages [20].

### 3.4. PGE_2_ Inhibits P2Y-Dependent Cell Migration

MEFs migration is affected by extracellular nucleotides [31]. As Figure 5(b) shows, transwell migration analysis of MEFs carrying a COX-2 transgene and maintained in the presence of DFU showed a response that was increased in cells treated with UTP, a process that was attenuated after pretreatment with PGE_2_. Interestingly, when PGE_2_ was present in both the upper and lower compartments, cell migration was completely abolished stressing the effect of this prostaglandin in the regulation of MEFs motility.

## 4. Comment

Extracellular nucleotides, such as UTP, have been described as innate immune regulators acting via the P2 receptors [32, 33]. Indeed, P2 agonists are increasingly viewed as a new class of innate immune system mediators following their release at sites of inflammation as a result of infection or cell damage [34]. Indeed, interplay between PGs and P2Y response in the context of macrophage activation, polarization, and resolution of the inflammation has been described [20]. However, less is known regarding the role of P2Y receptors and PGE_2_ in other cell types. For this reason, in this work, we provide new data on the fine regulation of P2Y signaling in MEFs using a specific agonist. Since fibroblasts play a role in the immune response [35], our data suggest that MEFs may play a central role in the regulation in the proresolution and tissue repair phase [36].

We have characterized the expression of P2Y2, P2Y4, and P2Y6 in MEFs, using functional and immunological approaches. Experiments were performed in MEFs from the WT and COX-2-deficient animals, carrying a COX-2 transgene. The release of PGE_2_ impaired UTP-dependent Ca^2+^-mobilization responses that could be attributed to the accumulation of PGE_2_ in the culture medium. These data clearly establish a regulation of P2Y receptors by PGE_2_ in MEF cells, in addition to other cells such as macrophages [20]. Interestingly enough, in the intact animal, this PGE_2_ can be derived by several COX-2 expressing cells acting in a concerted way. Moreover, it has been described that P2X7 receptor activation is required for the release of PGE_2_ in macrophages [37] which in turn could regulate P2Y responses. Taking this into account, it seems that there is a complex crosstalk between P2 receptors and PGE_2_ release.

All of the cloned P2Y receptors activate phospholipase C resulting in IP3 generation and Ca^2+^ release from intracellular stores. However, the response of the P2Y receptors is regulated by mechanisms involving desensitization that comprises phosphorylation of the receptors by protein kinases such as protein kinase C (PKC), attenuating receptor signaling [38]. Moreover, previous studies have demonstrated that P2Y receptors desensitization has been attributed to PKC-dependent mechanisms [39, 40]. In the present work, we have provided evidence for the involvement of PGE_2_, through PKC, in P2Y receptor desensitization, analyzing Ca^2+^ mobilization as read-out. We elucidated the main PKC isoenzyme responsible for the alterations of Ca^2+^ mobilization by choosing selective PKC inhibitors [30]. As controls, we used PGE_2_ and DFU, a selective COX-2 inhibitor which restores the UTP response, as in MEF WT and in KI cells, suggesting the regulation of P2 receptors signaling by PGE_2_. Also, we used Gö6976, for inhibiting the classic PKC isoforms, and Gö6850 that is structurally similar to the poorly selective PKC inhibitor staurosporine. Gö6850 shows high selectivity for PKC*α*, *β*1, *β*2, *γ*, *δ*, and *ε* isoenzymes [41]. Gö6983 is a pan-PKC inhibitor against PKC*α*, *β*, *γ*, and *δ*. Moreover, phorbol 12,13-dibutyrate (PDBu), a potent activator of PKC/PKD, and *α*-phorbol didecanoate (*α*PDD), which is an inactive derivate of PDBu, supported the role of these kinases in the regulation of P2Y activity by PGE_2_. Furthermore, PKDs regulate diverse cellular processes such as P2 signaling [26]. Previous data described that activation of PKC*δ* acts as an upstream PDK1 activation step [42]. For this reason, we use a selective PKD1 inhibitor, CID755376. Taken together, these data indicate that activation of PKC/PKD reduced Ca^2+^-mobilization by UTP. Using selective PKC and PKD inhibitors we hypothesized a key role for PKCs, although we cannot determine the specific isoforms involved in the alteration of Ca^2+^-mobilization by PGE_2_ after stimulation with UTP. The absence of effect after treatment with dibutyryl-cAMP indicates that the inhibition by PGE_2_ is independent of PKA. These conclusions agree with previous evidence describing a regulation of P2Y signaling by PGE_2_ [20]. Our data also indicate that the EP1–4 and P2Y receptors expression was not influenced by COX-2 activity. Interestingly, PGE_2_ did not affect Ca^2+^ fluxes by ionomycin but suppressed the effect of thapsigargin, suggesting that PGE_2_ alters Ca^2+^-mobilization from intracellular stores.

PGE_2_ promotes an internalization of P2Y4 in MEFs transfected with COX-2, an effect that is suppressed after inhibition of COX-2 with DFU. Based on these results, we hypothesize that the alteration in Ca^2+^-mobilization in response to UTP in MEFs transfected with COX-2 might be due to a lower membrane localization of P2Y4 when PGE_2_ production is enhanced. Moreover, the blockade in Ca^2+^-mobilization by PGE_2_ has an important reflect in terms of activation of different signaling pathways, including key regulators such as PKCs and energetic metabolism via AMPK activation and ACC inhibition.

Cell migration contributes to normal development and differentiation. Evidences in recent years have indicated that extracellular nucleotides can regulate the movement of “professional phagocytes” (macrophages, neutrophils, lymphocytes, and microglia) and other cell types (e.g., fibroblasts, endothelial cells, neurons, and keratinocytes) [43]. From a functional point of view, our data demonstrate that PGE_2_ inhibits P2Y-dependent cell migration, regardless of chemoattractant. These observations are in agreement with Koizumi et al. and other authors who described that P2Y2,4,6 receptors participate in chemotactic actions [44]. In this way, recent studies have focused on stromal cells, such as macrophages and fibroblasts, playing a role in the inflammatory lesion. Here we describe a cross-regulation between PGE_2_ and P2Y signaling that is independent of the PG receptors in MEFs. This mechanism is similar to that described by Través et al. [20], suggesting that macrophages and fibroblasts contribute to the regulation of inflammatory response and repair of tissue damage through aligned mechanisms involving P2Y signaling [35, 36]. Overall, the work suggests that targeting the stromal microenvironment is likely to be an important strategy for future anti-inflammatory therapies.

## Supplementary Material

Supplemental Figure S1. Video-imaging of Ca^2+^-dependent UTP-signaling in MEFS
Video 1: COX-2 KI cells were treated as indicated in the legend and the Ca^2+^ -response to UTP was analyzed.
Video 2: COX-2 KI cells were extensively washed with medium to remove the accumulated PGs in the extracellular medium (10-15 min of perfusion with Locke`s medium at 37°C) and challenged with UTP.
Video 3: COX-2 KI cells were maintained in the presence of DFU and, after perfusion with Locke's medium containing DFU, the Ca^2+^ -response to UTP was analyzed.
Video 4: COX-2 KI cells were detached from the dishes, and reseed for 90 min in the presence of DFU and, after perfusion with Locke's medium containing DFU, the Ca^2+^ -response to UTP was analyzed.
Video 5: WT cells treated as indicated in the legend and the Ca^2+^ -response to UTP was analyzed.
Video 6: WT cells were extensively washed with medium to compare with video 2 (10-15 min of perfusion with Locke`s medium at 37°C) and challenged with UTP.
Video 7: WT cells were perfused with Locke's medium containing PGE2 and the Ca^2+^ -response to UTP was analyzed.
